# Retracted: Prevalence and Associated Factors of Peer Victimization (Bullying) among Grades 7 and 8 Middle School Students in Kuwait

**DOI:** 10.1155/2019/8462304

**Published:** 2019-02-03

**Authors:** International Journal of Pediatrics

At the request of the authors, the article titled “Prevalence and Associated Factors of Peer Victimization (Bullying) among Grades 7 and 8 Middle School Students in Kuwait” [1] has been retracted. The study was conducted in agreement with the school Principal and the authors received verbal approval, but they did not receive formal ethical approval from the designated committee of the Ministry of Education.
